# Physical Exercise and Angiotensin II Receptors: A Systematic Review

**DOI:** 10.3390/biomedicines14091956

**Published:** 2026-08-30

**Authors:** Alice Miranda de Oliveira, Nataniele Santana de Souza, Lidiane Canuto Rodrigues, Ayala Sara Pereira de Oliveira, Pedro Elias Santos Souza, Ramon Martins Barbosa, Ana Marice Teixeira Ladeia, Jefferson Petto

**Affiliations:** 1 Postgraduate Department, Bahian School of Medicine and Public Health, Salvador 40290-000, Brazil; peedroefisio@gmail.com (P.E.S.S.); amonmartinsbarbosa@hotmail.com (R.M.B.);; 2 Actus-Cordios, Cardiovascular and Metabolic Rehabilitation, Salvador 40140-060, Brazil; 3 Paralela Campus, Ruy Barbosa Wyden University Center, Salvador 41730-101, Brazil; natani.san@gmail.com (N.S.d.S.); lidianerodrigues120@gmail.com (L.C.R.); 4 Evangelical Hospital of Cachoeiro de Itapemirim, Cachoeiro de Itapemirim 29308-020, Brazil; ayalasara14@gmail.com; 5 Health Sciences Center, Federal University of Recôncavo da Bahia, Santo Antônio de Jesus 44570-000, Brazil

**Keywords:** exercise, renin–angiotensin system, angiotensin receptors, angiotensin receptor type 1, angiotensin receptor type 2

## Abstract

**Background:** A functional balance between angiotensin II receptors (AT1R/AT2R) is associated with the regulation of cardiovascular, inflammatory, and tissue remodeling processes. Interventions that promote this balance have a significant impact on quality of life. Physical exercise stands out among these interventions, as the literature extensively identifies it as a therapeutic tool for managing conditions linked to the main axis of the renin–angiotensin system (RAS). Despite its beneficial role regarding the RAS, its effects on AT1R and AT2R receptor expression have not yet been systematically synthesized. **Objective:** To review the literature and systematically compile existing knowledge regarding the effect of physical exercise on the expression of angiotensin II receptors (AT1R and AT2R) within the RAS. **Methods:** This is a systematic review registered in PROSPERO (CRD420251267388). Searches were conducted in the PubMed/MEDLINE, PEDro, Cochrane Central Register of Controlled Trials, Scientific Electronic Library Online, Latin American and Caribbean Health Sciences Literature (LILACS), Web of Science, and Scopus databases. Risk of bias was assessed using the RoB 2 and SYRCLE tools. A total of 724 studies were identified, of which 11 met the eligibility criteria: 10 conducted using experimental animal models and 1 involving humans. **Results:** Eleven studies were included, predominantly animal experimental models. The findings indicate that chronic cyclic physical exercise is associated with reduced AT1R expression and increased AT2R expression in different tissues. In contrast, the only study that evaluated resistance exercise showed a distinct physiological response, with increased AT1R expression. **Conclusions:** The results suggest that chronic cyclical physical exercise promotes an increase in AT2R receptor expression and a reduction in AT1R expression in various body tissues, although the evidence is derived primarily from animal studies.

## 1. Introduction

The renin–angiotensin system (RAS) is one of the most extensively studied biological pathways in the scientific community, due to its numerous clinical applications, particularly in populations with cardiovascular dysfunction [1]. One of the key factors modulating its activity is the expression of Angiotensin II (Ang II) receptors, which are referred to as Angiotensin II type I and II receptors (AT1R and AT2R). The importance of these receptors in the effects triggered by the main axis of the RAS is well-established; they have long been studied for the development of drugs aimed at treating cardiovascular diseases and, more recently, conditions in cancer patients [1].

We know that the pathway that facilitates the binding of Ang II to AT1R, when overactivated, is closely linked to dysfunction in various organs, such as the heart and blood vessels [2]. A functional balance in the AT1R/AT2R ratio life is associated with fewer adverse health effects [3]. Mélissa Colin et al. [3] point out that the functional balance is determined by the balanced expression of these receptors, especially within the tissue-level RAS. Therefore, pharmacological or non-pharmacological interventions that promote this functional balance have significant impacts on quality of life.

Among non-pharmacological interventions, physical exercise stands out; it has been extensively studied and highlighted in the literature as a powerful therapeutic tool for managing various diseases directly linked to RAS dysfunction, such as systemic hypertension and heart failure. Some reviews highlight the extent to which and how physical exercise can act favorably and directly on the main axis of the renin–angiotensin system [4]. However, specifically regarding the effect of physical exercise on the expression of AT1R and AT2R, to the best of our knowledge to date, no studies have been published that systematically compile the results of research aimed at investigating the hypothesis that physical exercise modifies the expression of these receptors. Therefore, the aim of this scientific study was to review the literature and systematically compile existing knowledge on the effect of physical exercise on the expression of AT1R and AT2R receptors in the RAS.

## 2. Methods

### 2.1. Study Type

This systematic review was conducted and reported in accordance with the Preferred Reporting Items for Systematic Reviews and Meta-Analyses (PRISMA) 2020 statement [5]. The study was prospectively registered on the PROSPERO platform under registration number CRD420251267388. The review was structured according to the PICO framework, with the aim of answering the following question: in adult humans and/or animal models, what is the effect of physical exercise on the expression of angiotensin II type 1 (AT1R) and type 2 (AT2R) receptors?

The population (P) included adult humans (≥18 years) and experimental animal models; the intervention (I) consisted of physical exercise; the comparator (C) comprised sedentary groups, usual care, and/or no intervention; and the outcomes (O) involved the expression of AT1R and AT2R receptors.

### 2.2. Eligibility Criteria

Studies were considered eligible if they met the following criteria: (1) randomized or non-randomized clinical studies involving human participants, or controlled experimental studies conducted in animal models; (2) investigated the effects of a structured physical exercise intervention, including aerobic/endurance exercise, resistance exercise, or voluntary physical activity, on the expression of AT1R and/or AT2R; (3) included a comparison group (sedentary, sham, or control); and (4) reported receptor expression assessed by validated molecular or histological techniques. For human studies, participants were required to be adults aged ≥18 years. No restrictions were applied regarding sex, health condition, animal species, or tissue analyzed.

We excluded studies that did not use physical exercise as the primary intervention and studies that combined physical exercise with pharmacological interventions or other therapies when it was not possible to isolate the effect of exercise. We also excluded studies that did not provide an adequate description of the exercise protocol (intensity, duration, or frequency) and studies conducted in populations with conditions that significantly alter RAS regulation, such as acute systemic infections or active exposure to tobacco smoke, when these were not analyzed separately.

Because the objective of this review was to comprehensively evaluate exercise-induced changes in AT1R and AT2R expression across different biological systems, evidence from both human and controlled animal studies was considered eligible. Experimental animal models provide mechanistic information and permit tissue-specific analyses that are often not feasible in humans due to ethical and technical limitations. Therefore, the inclusion of both study types was considered essential to obtain a comprehensive understanding of the biological effects of exercise on the RAS.

### 2.3. Outcome of Interest

The primary outcome of this review was the expression of AT1R and AT2R receptors.

Receptor expression was considered eligible when assessed at the gene or protein level. Gene expression was assessed using reverse transcription polymerase chain reaction (RT-PCR) or quantitative PCR (qPCR), whereas protein expression was assessed using methods such as Western blotting, immunohistochemistry, or enzyme-linked immunosorbent assay (ELISA). Studies using other validated molecular or histological methods for the direct assessment of AT1R or AT2R expression were also considered eligible.

Data were extracted according to the receptor subtype (AT1R or AT2R), biological level assessed (mRNA or protein), tissue analyzed, and method used for receptor quantification.

### 2.4. Search Strategies

Two authors (A.M.O. and R.M.B.) independently conducted the electronic searches between August 2025 and 15 March 2026. The databases were searched from their inception until 15 March 2026, and included PubMed/MEDLINE, PEDro, the Cochrane Central Register of Controlled Trials (CENTRAL), Scientific Electronic Library Online (SciELO), Latin American and Caribbean Health Sciences Literature (LILACS) via the Virtual Health Library (VHL), Web of Science, and Scopus. The search strategies combined controlled vocabulary, including Medical Subject Headings (MeSH) and Health Sciences Descriptors (DeCS), with free-text terms related to physical exercise, the renin–angiotensin system, angiotensin II receptors (AT1R and AT2R), gene expression, and protein expression. Boolean operators (AND and OR) were used to combine search concepts. Database-specific search fields were applied according to the characteristics of each platform, including MeSH and field-specific searches in PubMed/MEDLINE, topic searches (TS) in Web of Science, and title, abstract, and keyword searches (TITLE-ABS-KEY) in Scopus. The search syntax was adapted to the indexing system and interface of each database. The complete search strategies, including the search terms, Boolean operators, and database-specific fields, are provided in Supplementary Table S1.

### 2.5. Searching Other Sources

In addition to searching electronic databases, we consulted the gray literature using Google Scholar to identify other published studies. We examined the direct citations of all the studies we included and of other relevant studies using Google Scholar (https://scholar.google.co.uk/) to obtain additional references; see Supplementary Table S2.

### 2.6. Study Selection and Data Extraction

Study selection was independently performed by two reviewers (A.M.O. and R.M.B.). All retrieved records were imported into Rayyan, where duplicate records were identified and removed before screening. Titles and abstracts were then independently screened according to the predefined eligibility criteria. Studies considered potentially eligible underwent full-text assessment to confirm their eligibility. Any disagreements regarding study selection were resolved through discussion, and when consensus could not be reached, a third reviewer (J.P.) made the final decision. In addition, the reference lists of all included studies were manually screened to identify potentially eligible studies that had not been retrieved through the electronic database searches. 

Data extraction was independently performed by two reviewers using a standardized data extraction form. The following information was collected from each included study: (1) author and year of publication; (2) study design; (3) population characteristics, including intervention or experimental and control groups; (4) method used to measure AT1R and AT2R expression (when both mRNA and protein expression were reported, these outcomes were extracted separately); (5) tissue evaluated; and (6) main results. Data extracted regarding the exercise protocol included: (1) type of exercise and equipment used; (2) exercise intensity; (3) session duration or dosimetry; (4) weekly frequency; and (5) program or protocol duration. Exercise protocol characteristics were also considered when interpreting the comparability of the included studies and the consistency of the findings across different exercise modalities, intensities, frequencies, and intervention durations.

### 2.7. Risk of Bias (Human Studies)

The risk of bias of the included randomized clinical trials was independently assessed by two reviewers using the Cochrane Risk of Bias 2 (RoB 2) tool [6]. This tool evaluates five domains related to the randomization process, deviations from intended interventions, missing outcome data, outcome measurement, and selection of the reported result. Each domain was classified as “Low Risk of Bias,” “Some Concerns,” or “High Risk of Bias,” and the overall judgment for each study was determined according to the highest-risk domain. Disagreements between reviewers were resolved through discussion until consensus was reached. 

### 2.8. Risk of Bias (Animal Studies)

Risk of bias in the included animal studies was independently assessed by two reviewers using the SYRCLE (Systematic Review Centre for Laboratory Animal Experimentation) risk-of-bias tool [7], which was specifically developed for preclinical studies. The tool evaluates 10 domains covering selection, performance, detection, attrition, reporting, and other sources of bias. Each domain was classified as low risk, high risk, or unclear risk of bias. Disagreements between reviewers were resolved by consensus. 

### 2.9. Data Summary

Because of substantial methodological and biological heterogeneity among the included studies, a formal meta-analysis was not performed. The included studies differed considerably in species and experimental models, exercise modality and dosage, tissue analyzed, receptor subtype assessed, biological level of measurement (mRNA or protein), and methods used to quantify AT1R and AT2R expression. These differences limited the comparability of the studies and precluded the estimation of a single clinically or biologically meaningful pooled effect.

Therefore, the findings were synthesized narratively and organized according to receptor subtype (AT1R and AT2R), exercise modality, biological level of assessment, tissue analyzed, and experimental model. The direction and consistency of the findings across studies were considered when interpreting the overall evidence, while divergent findings were explicitly reported rather than statistically pooled.

## 3. Results

**Figure 1:**
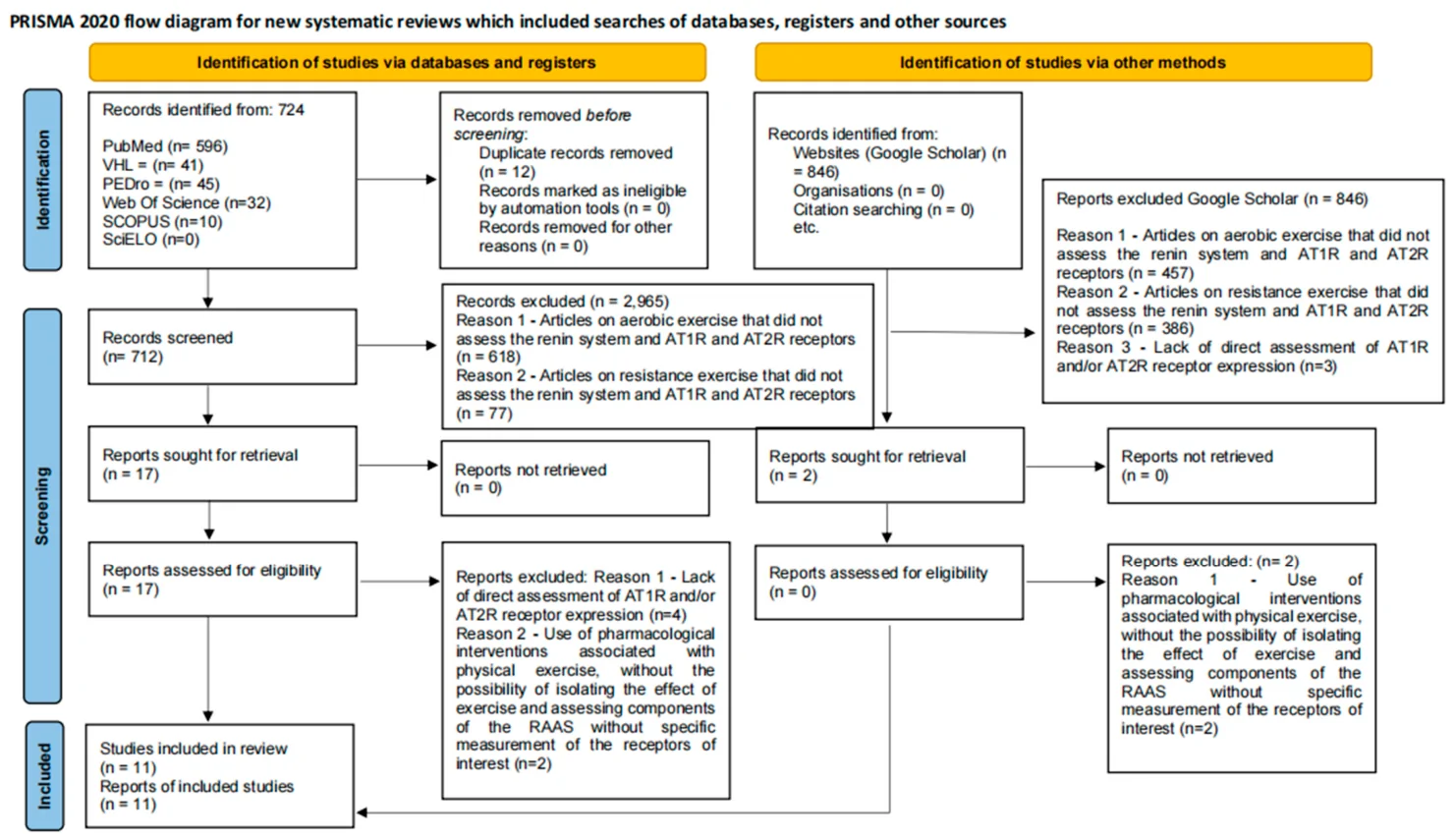
Flowchart illustrates the selection of studies included in the review (Source: Prepared by the authors).

We identified a total of 724 articles through the search strategies developed and the references reviewed via manual search. However, following review by the reviewers, 12 were excluded due to duplication, leaving 712 studies. After screening based on title and abstract, 12 studies were selected. In a subsequent step, following screening based on eligibility criteria, 1 study was excluded. The main reasons for exclusion were: lack of direct assessment of AT1R and/or AT2R receptor expression; use of pharmacological interventions combined with physical exercise, without the possibility of isolating the effect of exercise; and assessment of RAS components without specific measurement of the receptors of interest. Finally, 11 studies met the established selection criteria and are summarized in Figure 1.

Table 1 presents the data extracted from the 11 included studies. We can see that the year of publication ranged from 2005 to 2021, comprising one randomized clinical trial conducted in humans with coronary artery disease and ten controlled experimental studies performed in animal models (Wistar, Sprague-Dawley, and Zucker rats, and C57BL/6 mice). We highlight the predominance of preclinical evidence, using different pathophysiological models, including renovascular hypertension, heart failure, obesity, and chronic kidney disease. The outcomes analyzed consisted of gene and/or protein expression of AT1R and AT2R, conducted in a variety of tissues, such as cardiac, vascular, renal, and skeletal muscle tissue.

Table 2 shows that the exercise protocols employed were predominantly cyclic (aerobic) and performed on a cycle ergometer [8], in swimming [10,11], or on a treadmill [12,13,14,16,17,18]. Only one study used a resistance training protocol [9] and one used voluntary activity on a treadmill [15]. Exercise intensity ranged from low to moderate, generally prescribed based on the percentage of maximum functional capacity (MFC), peak oxygen uptake (VO_2_ peak), treadmill speed, or the percentage of one-repetition maximum (1RM). The interventions consisted of daily sessions of approximately 60 min, with a weekly frequency of 5 to 7 days, and total durations ranging from 3 to 12 weeks.

### 3.1. Modulation of AT1R and AT2R Expression by Physical Exercise

The results demonstrated a significant ability of chronic physical exercise to modulate the expression of AT1R and AT2R. Cyclic physical exercise was associated with a reduction in AT1R expression and an increase in AT2R expression in almost all studies [8,12,13,14,15,16,18], with the exception of a single experimental study in obese rats [11] that demonstrated a divergent response with a reduction in AT2R expression.

Furthermore, in specific contexts such as resistance exercise, an increase in AT1R receptor expression was observed [9], indicating variability in the physiological response depending on the type of exercise employed (Table 1).

### 3.2. Risk of Bias Assessment

**Figure 2:**
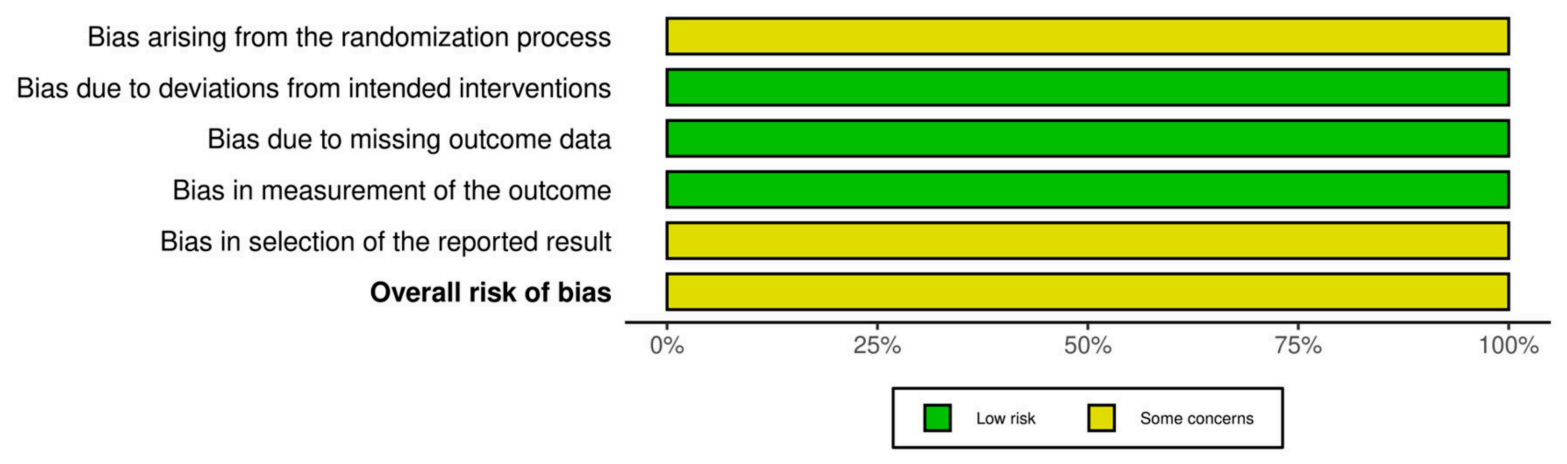
Results of the risk of bias assessment for the included human studies using the RoB 2 tool. Each domain was classified as low risk of bias (green) or some concerns (yellow), with the overall classification derived from these judgments.

**Figure 3:**
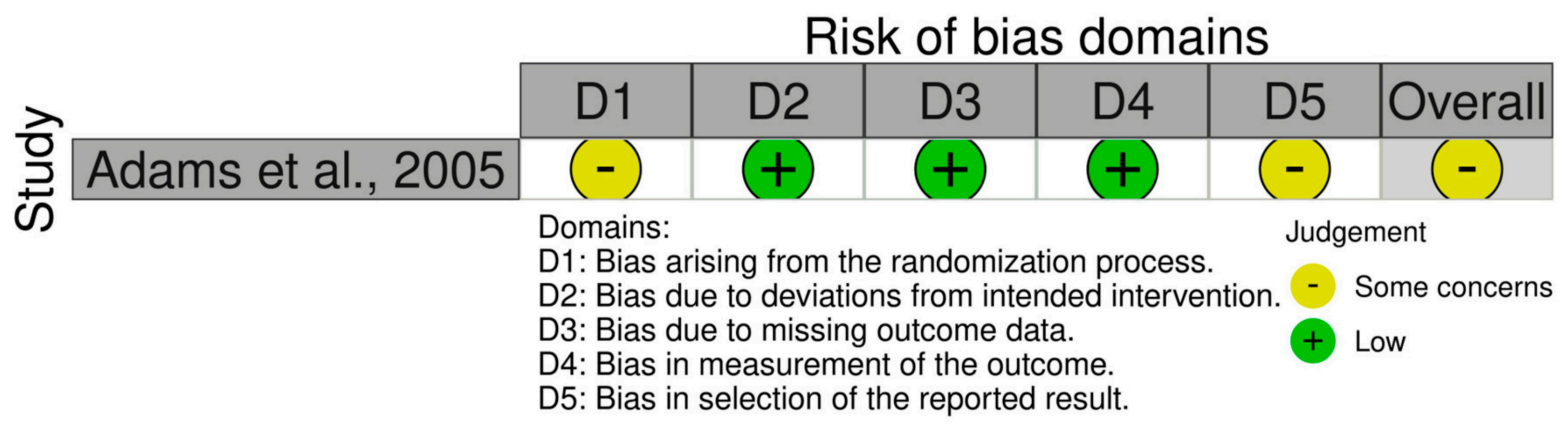
Risk of bias assessment for the included human studies using the RoB 2 tool. D denotes domain. D1, randomization process; D2, deviations from intended interventions; D3, missing outcome data; D4, measurement of outcomes; and D5, selection of the reported result. The color coding indicates low risk of bias (green) and some concerns (yellow) [8].

Regarding the risk of bias analysis (Figure 2 and Figure 3), the only human study included raised some points worthy of attention. This assessment is based primarily on the limited description of the randomization process and allocation concealment (Domain 1), as well as the absence of a pre-registered research protocol, which prevented verification of the selection of the reported outcome (Domain 5). However, the study demonstrated a low risk of bias in domains related to deviations from interventions (Domain 2), missing outcome data (Domain 3), and, notably, in outcome measurement, where assessor blinding was explicitly stated (Domain 4), lending robustness to the main findings despite the identified methodological concerns.

**Figure 4:**
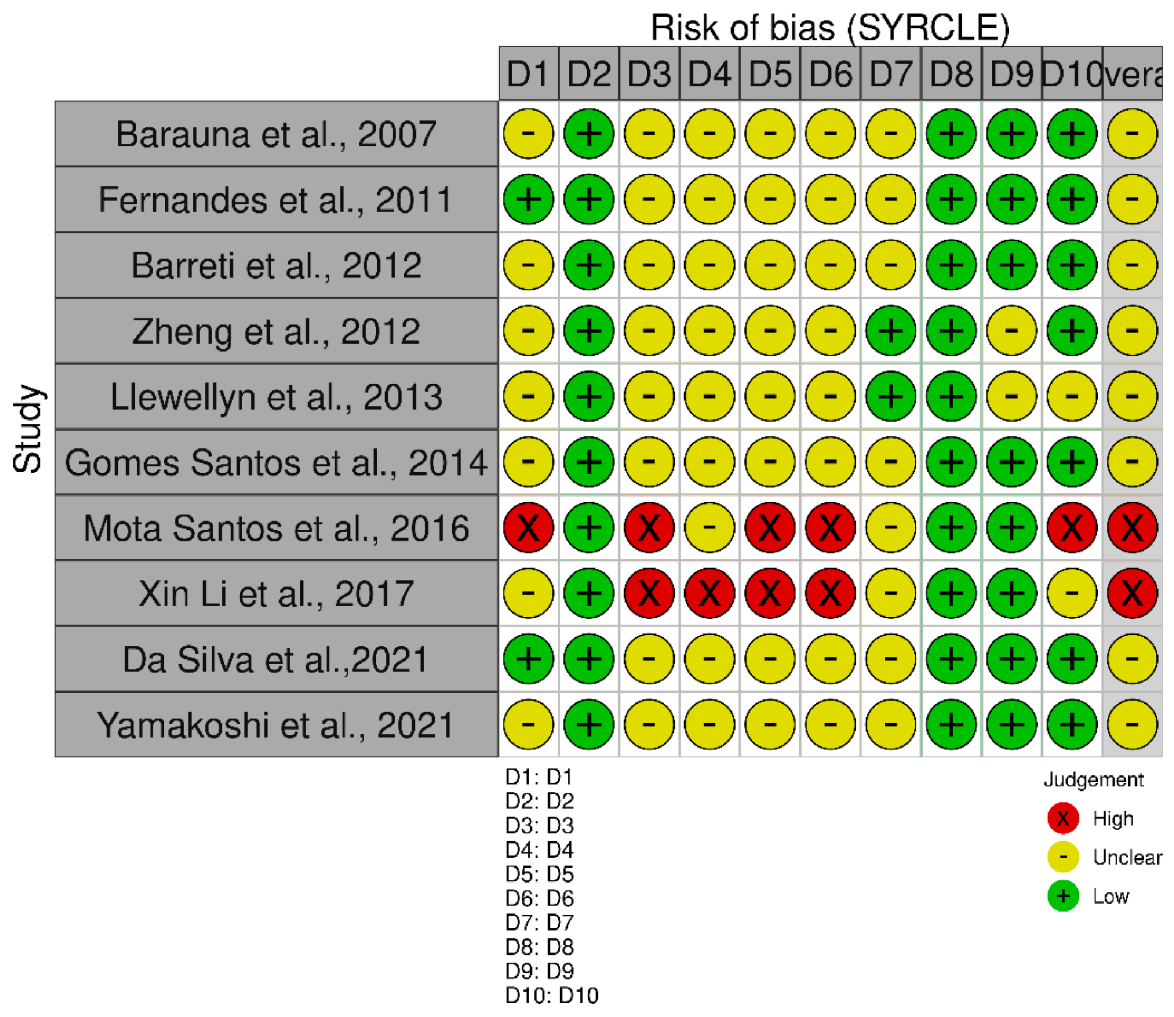
Results of the risk of bias assessment using the SYRCLE tool. D denotes domain. D1, sequence generation; D2, baseline characteristics; D3, allocation concealment; D4, randomized assignment; D5, blinding (performance bias); D6, randomized outcome assessment; D7, blinding (detection bias); D8, missing outcome data; D9, selective reporting of outcomes; and D10, other sources of bias. The color coding indicates low risk of bias (green), unclear risk of bias (yellow), and high risk of bias (red) [9,10,11,12,13,14,15,16,17,18].

**Figure 5:**
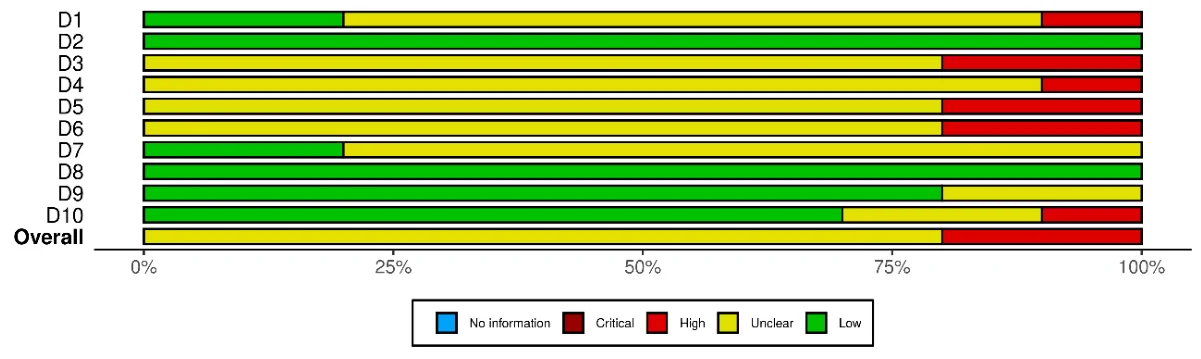
Results of the risk of bias assessment using the SYRCLE tool. D denotes domain. D1, sequence generation; D2, baseline characteristics; D3, allocation concealment; D4, randomized assignment; D5, blinding (performance bias); D6, randomized outcome assessment; D7, blinding (detection bias); D8, missing outcome data; D9, selective reporting of outcomes; and D10, other sources of bias. The color coding indicates low risk of bias (green), unclear risk of bias (yellow), high risk of bias (red), critical risk (orange), and no information (blue).

The assessment of risk of bias in experimental studies using the SYRCLE tool, as shown in Figure 4 and Figure 5, revealed a predominance of “unclear risk” classifications in domains that depended on detailed methodological descriptions related to randomization and blinding. Overall, the studies presented “low risk of bias” regarding baseline characteristics (D2) and the handling of incomplete outcome data (D8), indicating comparability between the experimental groups and appropriate management of sample losses. The domains related to selective reporting (D9) and other sources of bias (D10) were also predominantly classified as low risk. In contrast, the domains regarding random sequence generation (D1), allocation concealment (D3), randomization of participants (D4), performance blinding (D5), and random assignment of outcome assessment (D6) were mostly classified as “unclear risk,” primarily due to the lack of sufficient methodological information in the included studies.

### 3.3. Narrative Synthesis

A quantitative synthesis was not performed because the included studies showed substantial methodological and biological variability. The studies differed in animal species and experimental models, exercise modality and intensity, intervention duration, tissue analyzed, receptor subtype assessed, biological level of measurement (mRNA or protein), and methods used to quantify AT1R and AT2R expression. These differences limited the comparability of the findings and precluded the estimation of a single pooled effect.

Overall, most studies evaluating aerobic/endurance exercise reported reductions in AT1R expression and/or increases in AT2R expression. However, the magnitude and direction of the effects varied according to the experimental model, tissue, receptor subtype, and level of assessment. Divergent findings were also observed, including reduced AT2R expression in obese rats and increased AT1R expression following resistance exercise. The findings were therefore synthesized qualitatively according to receptor subtype, exercise modality, tissue, biological level of assessment, and experimental model rather than statistically pooled.

## 4. Discussion

Based on the data compiled in this review, we can suggest that chronic cyclical physical exercise increases AT2 receptor expression and decreases AT1 receptor expression. However, caution is required when interpreting the data, as virtually all the evidence supporting this inference comes from animal studies; while this strengthens the internal validity of the results, it raises questions regarding their extrapolation to humans. However, in the only study conducted on humans (a randomized clinical trial), the result was the same as that observed in animals: a reduction in AT1R expression and an increase in AT2R expression [8].

We should highlight the varied characteristics of the experimental groups studied, which ranged from healthy animals [9,10,15,16], animals with renovascular systemic hypertension [17], those with heart failure [12,13], and those with myocardial infarction [14]. In the human study, the population consisted of individuals with coronary artery disease [8]. Basically, in all these clinical conditions, the effect of exercise was the same, namely, increased AT2R expression and decreased AT1R expression. Only two studies showed a concomitant increase in both AT1R and AT2R [10,17] following exercise. However, the increase in AT2R was more pronounced than that of AT1R, with its elevation being two to three times greater (Table 1). Regarding the sample used in the studies, we observed heterogeneity among the animal species employed (male and female Wistar rats, Zucker rats, and male Sprague-Dawley rats). While this could introduce sampling bias, the experimental design—incorporating a control group across all studies—minimizes this confounding effect. Furthermore, we highlight that the increase in the AT2R receptor and the decrease in AT1R were independent of the species used (Table 1).

In this regard, it is worth noting a discrepancy between the findings of the vast majority of studies and those of the study conducted by Barretti et al., 2012 [11], using obese rats. The study in question evaluated the outcome in cardiac tissue and indicated that chronic cyclic exercise did not alter AT1R expression and decreased AT2R expression. The rationale provided by the authors for this divergent response is based on the clinical condition of the population (obesity). According to the authors, AT2R is elevated at baseline as a compensatory mechanism to maintain RAS balance, and the decrease in AT2R following training is beneficial for the AT1R/AT2R balance. We emphasize that this was the only study to present results and a physiological rationale distinct from those of the other studies.

Another relevant and distinctive aspect of the studies is that receptor expression was assessed in different tissues; that is, most studies sought to observe receptor increases along the tissue axis of the RAS. Among the tissues evaluated are: vascular endothelium [8], myocardium [9,10,11,15,16], active skeletal muscle [14,15], brain [12,13], renal cortex [18], and intestine [17]. In this regard, we might infer that the heterogeneity of the tissues evaluated could be a confounding factor. However, as noted in the preceding paragraph, only the study by Barretti et al. (2012) [11] reported divergent results, specifically regarding the myocardium. Yet, other authors who also evaluated receptor expression in the myocardium [10,15] observed a different response—namely, an increase in AT2R and a decrease in AT1R. Therefore, there currently appears to be no evidence that tissue-specific regulation interferes with the effect of physical exercise on AT1R and AT2R receptor expression.

There were two common points in the included exercise protocols: cyclic exercise and assessment of the effect of chronic exposure. Only one study evaluated the effect of resistance exercise. Therefore, the effects of physical exercise on AT1R and AT2R refer to chronic programs (lasting 3 to 10 weeks) that focused primarily on cyclic exercise. Furthermore, the weekly frequency ranged from 5 to 7 days per week, and the most commonly tested intensity was moderate. It is worth noting that only two studies used low-intensity exercise [10,11], but with a higher volume (180 min per day, five times per week), yielding the same results as the moderate-intensity protocols.

At this point in the discussion, a more specific approach to the only article that used resistance exercise is warranted. In that study, AT1R and AT2R expression was examined in the myocardial muscle of healthy rats, and the result was the opposite of that observed in studies with cyclic exercise; that is, there was an increase in AT1R (56% in gene expression and 31% in protein expression) without a percentage change in AT2R [9]. This increase was consistent with physiological myocardial hypertrophy. This result raises two questions: 1. Does the effect of resistance exercise with loads for the development of hypertrophy cause an increase in AT1R in all body tissues or only in those involved in force overload, such as the myocardium? 2. Is this increase in AT1R in the myocardium a physiological adaptation consistent with the need?

In light of the results presented in the studies cited here, it is imperative that we examine the mechanisms that may explain why physical exercise increases AT2R expression and decreases AT1R expression. Some studies do not explain the reasons for this adaptation [15,17]. Gomes-Santos et al., 2014 [14], however, suggest that exercise promotes a reduction in sympathetic activity, which stimulates a decrease in AT1R mRNA expression and an increase in AT2R mRNA expression. The hypothesis that elevated sympathetic activity directly contributes to increased RAS activity is supported by other studies conducted on groups with heart failure [19,20,21]. In fact, as observed in the study by Xin Li and Kun Wang, 2017 [16], the lower production of Angio II associated with lower AT1R expression in the hearts of rats with ischemic disease can also be explained by decreased sympathetic activity. This is because sympathetic activity stimulates renin production by the kidneys, which favors the main axis of the RAS by producing more Angio II, which in turn feeds back into AT1R activity and expression [22]. Thus, if exercise decreases sympathetic activity, it reduces the stimulus for renin production and consequently Angio II production, breaking this positive feedback loop.

Another hypothesis that may explain the change in AT1R and AT2R expression is mechanical muscle activity. According to Fernandes et al., 2011 [10], the improvement in the AT1R/AT2R expression ratio is linked to mechanical muscle stimulation, which promotes the production of Endothelial Growth Factor. It is worth noting that the authors demonstrated a concomitant increase in the expression of both receptors; however, the AT1R/AT2R ratio improves (with a greater increase in AT2R than in AT1R) following chronic cyclic exercise. This result was highlighted as an exercise-induced adaptation that tends to result in a better balance between the expression of these receptors. This leads us to the hypothesis that the decrease or increase in these receptors caused by exercise is adjusted to the physical demands imposed by the exercise and the clinical condition of the study group. This idea is corroborated by the article by Barauna et al., 2007 [9], which found an increase in AT1R and no change in AT2R when resistance exercise with loads was applied to induce hypertrophy. Note that, as mentioned earlier, the increase in AT1R in the myocardium did not result in pathological (concentric) hypertrophy, but rather in physiological hypertrophy necessary for the physical load imposed by exercise. However, it is worth reiterating that, although well-supported by the related scientific literature, the two mechanisms presented in the articles comprising this review are hypotheses that still require confirmation through mechanistic studies.

Finally, in light of the above, it is reasonable to believe that a chronic cyclic exercise program can benefit a wide range of patients, particularly those with cardiovascular diseases, by improving the AT1R/AT2R ratio. To cite some examples, patients with hypertension, heart failure, ischemic diseases, and other conditions have a high potential to benefit. We emphasize that for a condition such as cardiac cachexia, resistance exercise may be the best option, as it may promote physiological hypertrophy [9]. Further studies on resistance exercise should be conducted to determine whether this form of exercise can positively impact the reversal or reduction in cardiac cachexia. In summary, this may be the first systematic review on this topic, and we note that it is a field of experimental and clinical research with promising results, but one that still holds great potential for advances in scientific knowledge that could directly impact large populations, such as those with cardiovascular diseases.

### Limitations

This review presents certain limitations that should be considered when interpreting its findings. First, the majority of available evidence stemmed from experimental animal studies, with only one study conducted in humans identified; this limits the direct extrapolation of these results to human physiology or clinical practice.

Second, considerable methodological and biological heterogeneity was observed among the included studies, encompassing differences in species and experimental models; exercise modality, intensity, duration, and dosage; tissues analyzed; receptor subtypes evaluated; and laboratory techniques employed. Additionally, the inclusion of studies assessing receptor expression at different biological levels (mRNA and protein) warrants caution, as changes in gene expression do not always correspond proportionally to changes in protein expression, and the lack of methodological standardization hinders direct comparison between these measures. Due to this heterogeneity, a meta-analysis was not deemed methodologically appropriate; instead, the findings were synthesized using a narrative approach. Furthermore, the limited number of studies investigating resistance exercise and specific tissues constrains the robustness of conclusions regarding these specific conditions. Another significant limitation concerns the absence of a clear definition regarding the magnitude of change in receptor expression that would be considered clinically relevant, complicating the interpretation of the findings’ clinical significance. Confidence in the available evidence is also diminished by methodological limitations identified during the risk-of-bias assessmentspecifically, the incomplete description of randomization and blinding procedures in several experimental studies.

Finally, although excluding studies that combined physical exercise with pharmacological interventions allowed for a more specific assessment of exercise’s effects on AT1R/AT2R receptor expression, this criterion may have reduced the scope of the available evidence. Thus, the results of this review should be interpreted with caution and reinforce the need for well-designed clinical studiesusing standardized exercise protocols and harmonized methods for assessing receptor expressionto strengthen the translational relevance of the currently available evidence.

## 5. Conclusions

The findings of this review suggest that low- or moderate-intensity cyclic exercise, when performed chronically, reduces AT1R expression and increases AT2R expression in various body tissues. Two possible mechanisms underlie this effect: decreased sympathetic activity and mechanical muscle activity. However, caution is required in accepting such a conclusion, given that the results are based primarily on experimental studies involving animals.

## Figures and Tables

**Table 1:** Characteristics of the included studies and expression of the AT1R and AT2R subtypes following exercise programs.

Author, Year	Study Type	Population/Sample	Measurement Method	Tissue Evaluated	Results
Adams et al., 2005 [8]	Randomized Clinical Trial	Men with coronary artery disease (n = 45) divided into: • GI (n = 22): Mean age 63 ± 2 years; • GC (n = 23): Mean age 63 ± 2 years	Gene Expression (mRNA): Quantitative RT-PCR; Protein Expression: Western blot	Vascular: Internal mammary artery	AT1R: Gene expression (mRNA) was 77% lower in GI compared to GC. Protein expression was 46% lower in GI. AT2R: Gene expression (mRNA) was approximately 5 times higher in GI compared to GC. No difference in protein expression.
Barauna et al., 2007 [9]	Controlled Experimental	Healthy and normotensive Male Wistar rats (n = 36) divided into: • GC (n = 6); • GC + losartan (n = 6); • GC + high-salt diet (n = 6); • TFR (n = 6); • TFR + losartan (n = 6); • TFR + high-salt diet (n = 6)	Gene Expression (mRNA): Real-time RT-PCR; Protein Expression: Western blot	Cardiac: Left ventricle	AT1R(a): 56% increase in gene expression and 31% in protein expression in the TFR group compared to GC. AT2R: No significant changes.
Fernandes et al., 2011 [10]	Controlled Experimental	Normotensive Female Wistar rats (n = 21) divided into: • GS (n = 7): Sedentary; • G1 (n = 7): Low intensity and moderate volume; • G2 (n = 7): Low intensity and high volume	Gene Expression (mRNA): Quantitative RT-PCR	Cardiac: Myocardium	AT1R: Increase of 69% and 99% in G1 and G2, respectively. No modification in GS. Statistical difference detected only between trained groups and GS. AT2R: Increase of 26% and 332% in G1 and G2, respectively. No modification in GS. Statistical difference detected between G2 vs. GS and G2 vs. G1. No difference between G1 and GS.
Barretti et al., 2012 [11]	Controlled Experimental	Male Zucker rats (n = 20) divided into: • Lean Zucker (n = 5); • Lean Zucker + TF (n = 5); • Obese Zucker (n = 5); • Obese Zucker + TF (n = 5)	Protein Expression: Western blot	Cardiac: Left ventricle	AT1R: No significant changes. AT2R: In the obese Zucker + TF group, there was a 35% reduction in AT2 expression compared to the obese group without TF.
Zheng et al., 2012 [12]	Controlled Experimental	Male Sprague-Dawley rats divided into: • GS Sed (n = 8): Sham surgery Sedentary; • IC Sed (n = 8): With heart failure Sedentary; • GS TF (n = 8): Sham surgery and Physical Training; • IC TF (n = 8): With heart failure and Physical Training	Protein Expression: Western blot	Cerebral: Paraventricular nucleus	AT1R: AT1 receptor expression in the IC TF group significantly decreased, comparable to GS levels. AT2R: Not evaluated.
Llewellyn et al., 2014 [13]	Controlled Experimental	Male Sprague-Dawley rats divided into: • GS Sed (n = 8): Sham surgery Sedentary; • IC Sed (n = 8): With heart failure Sedentary; • GS TF (n = 8): Sham surgery and Physical Training; • IC TF (n = 8): With heart failure and Physical Training	Gene Expression (mRNA): RT-PCR TriReagent; Protein Expression: Western blot	Cerebral: Subfornical organ	AT1R: mRNA and protein expression of the AT1 receptor in the subfornical organ decreased in the IC TF group compared to IC Sed, returning to levels observed in the sedentary control group (GS Sed). AT2R: Not evaluated.
Gomes-Santos et al., 2014 [14]	Controlled Experimental	Male Wistar rats with induced myocardial infarction (n = 44) divided into: • GS (n = 10): Sham MI Sedentary; • G1 (n = 10): Sham MI Exercise; • G2 (n = 12): MI with HF Sedentary; • G3 (n = 12): MI with HF Exercise	Gene Expression (mRNA): Quantitative RT-PCR	Skeletal Muscle: Soleus and Plantaris.	AT1R: Lower gene expression in the soleus muscle only in group G3 compared to G2. AT2R: No difference detected.
Motta-Santos et al., 2016 [15]	Controlled Experimental	C57BL/6 mice divided into four groups: • GS (n = 8): Healthy without exercise; • G1 (n = 8): Healthy with exercise; • G2 (n = 8): With gene deletion of ACE2 production and sedentary; • G3 (n = 8): With gene deletion of ACE2 production with exercise	Gene Expression (mRNA): Quantitative RT-PCR	Cardiac: Myocardium; Skeletal Muscle: Gastrocnemius	AT1R: No difference in cardiac and skeletal gene expression was observed between groups. AT2R: Cardiac gene expression was higher in the ACE2 deletion exercise group compared to the ACE2 deletion exercise group. The sedentary group with ACE2 deletion also showed higher gene expression than the ACE2 deletion exercise group. No difference in skeletal gene expression was observed between groups.
Xin Li and Kun Wang, 2017 [16]	Controlled Experimental	Male Sprague-Dawley rats (n = 40) divided into: • GC—Healthy without PT (n = 20); • GE—Healthy with PT (n = 20)	Gene Expression (mRNA): Quantitative RT-PCR	Cardiac: Coronary artery smooth muscle	AT1R: Reduction in expression only in the exercise group. AT2R: Not evaluated.
Da Silva et al., 2021 [17]	Controlled Experimental	Male Wistar rats divided into (n—not informed): • GS—Healthy sedentary rats (Sham); • GC—Renovascular Hypertension rats (surgical induction) Sedentary; • GI—Renovascular Hypertension rats (surgical induction) Exercised	Gene Expression (mRNA): Quantitative RT-PCR.	Intestinal: Duodenum smooth muscle.	AT1R(a): No difference between groups. AT1R(b): Gene expression was 1x higher in GI than in GS and GC. AT2R: Gene expression was 2x higher in GI than in GS and GC.
Yamakoshi et al., 2021 [18]	Controlled Experimental	Male Sprague-Dawley rats divided into: • GS: Sham surgery (n = 7); • Nephrectomy + sedentary group (n=9); • GE: Nephrectomy + PT (n = 8)	Protein Expression: Western blot	Renal: Renal cortex	AT1R: PT did not significantly affect AT1R expression increased by nephrectomy. AT2R: Nephrectomy decreased AT2R expression. PT restored the decreased AT2R expression by nephrectomy.

Abbreviations: Ang II: Angiotensin II; AT1R: Angiotensin 1 Receptor; AT2R: Angiotensin 2 Receptor; AT1R(a): Angiotensin 1 Receptor type a; AT1R(b): Angiotensin 1 Receptor type b; ACE: Angiotensin-Converting Enzyme; GC: Control Group; GE: Experiment Group (Physical Training); GI: Intervention Group (Physical Training); GS: Sham Group; HF: Heart Failure; MI: Myocardial Infarction; RT-PCR: Reverse Transcription Polymerase Chain Reaction; PT: Physical Training; RPT: Resistance Physical Training; Sed: Sedentary.

**Table 2:** Description of the exercise programs used in the included studies.

Author, Year	Modality/ Equipment	Intensity	Session Duration or Dosimetry	Weekly Frequency	Program Duration
Adams et al., 2005 [8]	Cyclic/ Rowing and cycling ergometer	≥50 Watts	10 min, 3 times a day for each ergometer, totaling 60 min per day	7 days	4 weeks
Barauna et al., 2007 [9]	Resistance/ Squat simulator with electrical stimulation in the tail (vertical squat apparatus)	65–75% of 1RM	4 sets of 12 repetitions per day	5 days	8 weeks
Fernandes et al., 2011 [10]	Cyclic/ Swimming pool for rats	Low Intensity	Group 1—60 min per day; Group 2—60 min per day for the first 8 weeks; 120 min in the 9th week (60 min 2x a day); 180 min in the 10th week (60 min 3x a day)	5 days	10 weeks
Barretti et al., 2012 [11]	Cyclic/ Swimming apparatus with caudal dumbbells weighing 5% of body weight	Low Intensity	60 min per day	5 days	10 weeks
Zheng et al., 2012 [12]	Cyclic/ Motorized treadmill	Moderate Intensity; Speed of 20–25 m/min; Inclination of 5–10%	60 min per day	Not reported	3 weeks
Llewellyn et al., 2013 [13]	Cyclic/ Motorized treadmill	Moderate Intensity; Speed of 20–25 m/min; Inclination of 5–10%	60 min per day	5 days	3 to 4 weeks
Gomes-Santos et al., 2014 [14]	Cyclic/ Treadmill for rats	60% of peak VO2 of MFC	Exercise groups—60 min per day; Sham groups—5 daily min	5 days	8 weeks
Motta-Santos et al., 2016 [15]	Cyclic/ Voluntary activity wheel for mice	Not measured	Only reported that the number of daily turns on the voluntary activity wheel was 30% in the ACE2 deletion exercise group compared to the healthy exercise group	7 days	6 weeks
Xin Li and Kun Wang, 2017 [16]	Cyclic/ Treadmill for rats	60 to 65% of MFC	Starting with 15 evolving to 50 daily min	6 days	8 weeks
Da Silva et al., 2021 [17]	Cyclic/ Treadmill for rats	55 to 65% of MFC	60 min per day	5 days	6 weeks
Yamakoshi et al., 2021 [18]	Cyclic/ Treadmill for rats	Moderate Intensity; 20 m/min, no inclination	60 min per day	5 days	12 weeks

Abbreviations: MFC: Maximum Functional Capacity; VO2 peak: Peak Oxygen Volume; ACE2: Angiotensin-Converting Enzyme 2; 1RM: 1 Repetition Maximum.

## Data Availability

No new data were created or analyzed in this study. Data sharing is not applicable to this article.

## Supplementary Materials

The following supporting information can be downloaded at: https://www.mdpi.com/article/10.3390/biomedicines14091956/s1. Supplementary Table S1. Search strategies used in the databases. Supplementary Table S2. Google Scholar Search Strategies.

## Abbreviations

The following abbreviations are used in this manuscript:

Ang II	Angiotensin II
AT1R	Angiotensin type I receptors
AT2R	Angiotensin type and II receptors
RAS	Renin–angiotensin system
