# Supplementary material for: Physical Exercise and Angiotensin II Receptors: A Systematic Review
Source: Biomedicines. 2026 Aug 30;14(9):1956. doi: 10.3390/biomedicines14091956 (PMC13604229; doi:10.3390/biomedicines14091956)
Supplement: Supplementary file 1 [file biomedicines-14-01956-s001.zip › biomedicines-4407997-supplementary.pdf]

Supplementary Table S1. Search strategies used in the databases.

**Pubmed/Medline**	("Exercise"[MeSH Terms] OR "Exercise Therapy"[MeSH Terms] OR "Physical Activity"[All Fields] OR "Exercise Training"[All Fields] OR "Aerobic Exercise"[All Fields] OR "Resistance Training"[All Fields]) AND ("receptors, angiotensin"[MeSH Terms] OR "Angiotensin II Type 1 Receptor"[All Fields] OR "AT1R"[All Fields] OR "Angiotensin II Type 2 Receptor"[All Fields] OR "AT2R"[All Fields] OR "Angiotensin receptor"[All Fields])
	("exercise"[MeSH Terms] OR "exercise"[All Fields] OR "exercises"[All Fields] OR "exercise therapy"[MeSH Terms] OR ("exercise"[All Fields] AND "therapy"[All Fields]) OR "exercise therapy"[All Fields] OR "exercising"[All Fields] OR "exercises"[All Fields] OR "exercised"[All Fields] OR "exerciser"[All Fields] OR "exercisers"[All Fields]) AND ("receptor angiotensin type 1"[Supplementary Concept] OR "receptor angiotensin type 1"[All Fields] OR "receptor, angiotensin, type 1"[MeSH Terms] OR "type 1 angiotensin receptor"[All Fields]) AND ("receptor angiotensin type 2"[Supplementary Concept] OR "receptor angiotensin type 2"[All Fields] OR "receptor, angiotensin, type 2"[MeSH Terms] OR "type 2 angiotensin receptor"[All Fields])
	("Exercise"[MeSH Terms] OR "Exercise Therapy"[MeSH Terms] OR "Exercise"[Title/Abstract] OR "physical activity"[Title/Abstract] OR "exercise training"[Title/Abstract] OR "aerobic exercise"[Title/Abstract] OR "resistance training"[Title/Abstract]) AND ("receptors, angiotensin"[MeSH Terms] OR "Angiotensin II Type 1 Receptor"[Title/Abstract] OR "Angiotensin II Type 2 Receptor"[Title/Abstract] OR "AT1R"[Title/Abstract] OR "AT2R"[Title/Abstract] OR "angiotensin receptor"[Title/Abstract]) AND ("Gene Expression"[MeSH Terms] OR "Protein Expression"[Title/Abstract] OR "mRNA"[Title/Abstract] OR "Protein Expression"[Title/Abstract])
**Portal Regional da BVS / LILACS**	(tw:("exercício" OR "atividade física" OR "treinamento físico" OR "exercise" OR "physical activity" OR "exercise training" OR "aerobic exercise" OR "resistance training")) AND (tw:("receptores de angiotensina" OR "receptor de angiotensina tipo 1" OR "receptor de angiotensina tipo 2" OR "angiotensin receptors" OR "AT1R" OR "AT2R")) AND (tw:("expressão gênica" OR "expressão proteica" OR "gene expression" OR "protein expression" OR "mRNA"))
**Scielo**	(exercício OR "atividade física" OR "treinamento físico" OR exercise OR "physical activity") AND ("receptores de angiotensina" OR "angiotensin receptors" OR AT1R OR AT2R) AND ("expressão gênica" OR "expressão proteica" OR "gene expression" OR "protein expression" OR mRNA)
**PEDro**	exercise angiotensin / exercise AT1R / exercise AT2R
**Web of Science**	TS=(exercise OR "physical activity" OR "exercise training" OR "aerobic exercise" OR "resistance training") AND TS=("angiotensin receptor" OR "AT1R" OR "AT2R" OR "angiotensin II type 1 receptor" OR "angiotensin II type 2 receptor") AND TS=("gene expression" OR "protein expression" OR mRNA)

**SCOPUS**	TITLE-ABS-KEY (exercise OR "physical activity" OR "exercise training" OR "aerobic exercise" OR "resistance training") AND TITLE-ABS-KEY ("angiotensin receptor" OR "AT1R" OR "AT2R" OR "angiotensin II type 1 receptor" OR "angiotensin II type 2 receptor") AND TITLE-ABS-KEY ("gene expression" OR "protein expression" OR mRNA)

**Supplementary Table S2.** Google Scholar Search Strategies
(1) "exercise training" AND "renin angiotensin system" AND AT1R AND AT2R;<br>(2) "exercise AND angiotensin receptors" AND "AT1 receptor" AND "AT2 receptor";<br>(3) "exercise training" AND angiotensin receptor expression AND AT1R AND AT2R;<br>(4) "exercise training" AND angiotensin receptors AND rats OR mice OR humans.
